# Medical Legislation

**Published:** 1896-12

**Authors:** H. A. Tutwiler

**Affiliations:** Flatonia, Texas


### Correspondence.

### Medical Legislation.

FLATONIA, TEXAS, November 10, 1896.

*Editors Texas Medical Journal:*

The time for the next legislature to meet is fast approaching. Your JOURNAL wields a strong influence in our State, not only among medical men, but also, to a certain extent, among the laymen. *Will* you call the attention of the powers that be, both legislative and executive, to a class of persons in our State who are as much to be pittied as the leper or the criminal? I allude to the *epileptic*. Last year, in my address, when I had the honor to be president of the West Texas Medical Association, I called attention to this matter, and had intended to prepare statistics concerning them, their number, condition, etc. Unfortunately, a long spell of sickness prevented my doing so. And I was only able to refer to what is being done in their behalf at Senega, N. Y. This was at our annual meeting in October, 1895. When the State Medical Association met in April, 1896, the subject was also brought up, and I believe a committee appointed to urge the matter before the next legislature. I

quote (from memory) the principal remarks made by me in the address:

"These people are more wretched, and less account taken of their condition than any of the unfortunate who appeal to our sympathy or to legislative action. We have homes, reformatories, asylums and work shops for every class of the unfortunates in our State, except the epileptics. They, by the time they have reached middle age, have spent their all in the vain effort to get relief. Then they are sent to our poor houses, our jails, and even, sometimes, to the asylums for the insane. Many of them are people of refinement and of education, and only ask the privilege of being permitted to work. Who will hire them? The very nature of their malady debars them from every employment, no matter how humble. The State of Texas must keep up with the civilization of the nineteenth century. She must do what has been done in Germany, in New York and in one or two other States of our Union. Let her purchase five thousand acres of good land. Let her establish a colony, where all sorts of work can be done; where they can have homes and gardens. In a few years such an establishment would become self-supporting. Let there be medical men of eminence in that branch, appointed, with sufficient salary to give up everything else for that work, and that alone. Let them hold their positions for life or good behavior. Many of these people might be cured; at any rate, they would soon be no longer a burden, but would be more than self-supporting."

Now, if you can do anything to further this idea (or one better) do it. I have corresponded with and spoken to eight or ten members of our next legislature, and each and every one of them has promised me to do what he could.

Yours truly, H. A. TUTWILER, M. D.

